# Complete Blood Count-Derived Inflammatory Indices Following Whole-Body Cryotherapy Combined with Kinesiotherapy Versus Kinesiotherapy Alone in Men with Ankylosing Spondylitis

**DOI:** 10.3390/jcm15207894

**Published:** 2026-10-13

**Authors:** Agata Stanek, Ireneusz Ryszkiel, Ewa Romuk, Agnieszka Kiełboń, Armand Cholewka

**Affiliations:** 1 Department of Internal Medicine, Metabolic Diseases and Angiology, Faculty of Health Sciences in Katowice, Medical University of Silesia, Ziołowa 45/47, 40-635 Katowice, Poland; ryszkielirek@gmail.com; 2 Department of Biochemistry, Faculty of Medical Sciences in Zabrze, Medical University of Silesia, Jordana 19 St., 41-808 Zabrze, Poland; eromuk@sum.edu.pl; 3 Faculty of Science and Technology, University of Silesia in Katowice, 75 Pułku Piechoty 1A St., 41-500 Chorzów, Poland; agnieszka.kielbon@us.edu.pl (A.K.); armand.cholewka@us.edu.pl (A.C.)

**Keywords:** whole-body cryotherapy, complete blood count-derived inflammatory indices, systemic immune-inflammation index, systemic inflammation response index, neutrophil-to-lymphocyte ratio, platelet-to-lymphocyte ratio, monocyte-to-lymphocyte ratio, ankylosing spondylitis

## Abstract

**Background/Objectives:** The response of complete blood count (CBC)-derived inflammatory indices to whole-body cryotherapy (WBC) in ankylosing spondylitis (AS) remains unknown. This exploratory post hoc secondary analysis examined whether adding WBC to kinesiotherapy was associated with differences in these indices and explored their associations with disease activity, pain, and high-sensitivity C-reactive protein (hsCRP) levels. Prospectively collected data from 32 men with AS were analyzed. Participants were allocated by alternation, rather than randomization, to WBC combined with kinesiotherapy (WBC + K; *n* = 16) or kinesiotherapy alone (KT; *n* = 16). **Methods:** Prospectively collected data from 32 men with ankylosing spondylitis (AS) who participated in a prior study were analyzed. Participants were allocated to whole-body cryotherapy combined with kinesiotherapy (WBC + K; *n* = 16) or kinesiotherapy alone (KT; *n* = 16). Complete blood count parameters, high-sensitivity C-reactive protein (hsCRP) levels, Bath Ankylosing Spondylitis Disease Activity Index (BASDAI), and visual analogue scale (VAS) pain scores were assessed prospectively before and after the intervention. Previously unpublished complete blood count data were used to calculate the neutrophil-to-lymphocyte ratio (NLR), platelet-to-lymphocyte ratio (PLR), monocyte-to-lymphocyte ratio (MLR), systemic immune-inflammation index (SII), and systemic inflammation response index (SIRI) post hoc and to explore their associations with hsCRP, BASDAI, and VAS pain. The primary intervention effects were assessed using baseline-adjusted analysis of covariance, and the five treatment-effect *p*-values were subjected to Benjamini–Hochberg correction. Comparisons of changes from baseline were performed as sensitivity analyses. **Results:** None of the baseline-adjusted between-group treatment effects for NLR, PLR, MLR, SII, or SIRI were statistically significant before or after false discovery rate correction. The adjusted between-group difference for MLR was −0.033 (95% confidence interval: −0.074 to 0.008; *p* = 0.115; *q* = 0.329). In the change-score sensitivity analysis, the between-group difference in MLR change was nominally significant (*p* = 0.012) but did not meet the multiplicity-adjusted significance threshold (*q* = 0.060). Within the WBC + K group, PLR, MLR, and SIRI decreased significantly, whereas no corresponding within-group changes were observed in the KT group; however, these within-group findings were descriptive and did not establish an additional effect of WBC. Changes in the indices were not significantly correlated with changes in hsCRP, BASDAI, or VAS pain. None of the exploratory post-treatment correlations remained significant after multiple-testing correction. **Conclusions:** This study did not provide baseline-adjusted or multiplicity-adjusted evidence that adding WBC to kinesiotherapy affected any of the investigated CBC-derived inflammatory indices in men with AS. The nominal MLR signal identified in the change-score sensitivity analysis and the within-group reductions in PLR, MLR, and SIRI should be considered exploratory and hypothesis-generating. Larger, prospectively designed, adequately powered randomized studies with prespecified outcomes and baseline-adjusted analyses are required.

## 1. Introduction

Ankylosing spondylitis (AS) is a chronic immune-mediated inflammatory disease that primarily affects the sacroiliac joints and axial skeleton [1]. AS is estimated to affect approximately 1 in 200 individuals worldwide, although prevalence varies substantially across geographical and ethnic populations, partly reflecting differences in HLA-B27 distribution, diagnostic criteria, and study methodology. The number of individuals with AS has been estimated at 1.30–1.56 million in Europe and 4.63–4.98 million in Asia. Robust population-based estimates specifically for Poland are currently lacking; nevertheless, AS represents an important chronic rheumatic disease in the Polish population [2]. Persistent inflammation may lead to progressive structural damage, spinal stiffness, restricted mobility, and impaired physical function. In more advanced disease, these changes can result in substantial functional limitations and disability, reduced work participation, and deterioration in health-related quality of life [1,3,4]. Because AS commonly begins in early adulthood and follows a chronic course, its physical, psychological, social, and occupational consequences may persist for many years [5,6].

The management of AS requires a comprehensive approach combining pharmacological and non-pharmacological interventions. Pharmacological treatment has advanced considerably with the introduction of biological disease-modifying antirheumatic drugs targeting tumor necrosis factor and interleukin-17, as well as targeted synthetic therapies. Nevertheless, even in the era of biological treatment, regular exercise and rehabilitation remain fundamental components of disease management. The current ASAS–EULAR recommendations emphasize patient education, regular exercise, and consideration of physiotherapy as integral elements of care alongside pharmacological treatment [7]. Exercise-based rehabilitation aims to maintain spinal mobility, posture, muscle strength, cardiorespiratory fitness, and functional independence and may also reduce pain and stiffness. Supervised physiotherapy can be particularly useful in patients who require individualized exercise prescription or support with adherence. Thus, non-pharmacological treatment should be considered complementary to, rather than replaced by, advances in pharmacotherapy [7,8].

Accurate assessments of inflammatory activity and treatment response in AS remain challenging. Clinical indices, such as the Bath Ankylosing Spondylitis Disease Activity Index (BASDAI), capture the patient’s perception of symptoms, whereas conventional acute-phase reactants, including C-reactive protein (CRP), may remain within the reference range despite clinically active disease [9]. Consequently, increasing attention has been directed toward inexpensive inflammatory indices calculated from routine complete blood count parameters. These include the neutrophil-to-lymphocyte ratio (NLR), platelet-to-lymphocyte ratio (PLR), monocyte-to-lymphocyte ratio (MLR), systemic immune-inflammation index (SII), and systemic inflammation response index (SIRI). By integrating changes in neutrophils, lymphocytes, monocytes, and platelets, these indices may provide complementary information on the balance between systemic inflammation and immune regulation [9,10,11,12,13].

Previous studies have demonstrated associations of NLR, PLR, MLR, SII, and SIRI with inflammatory activity, BASDAI, CRP, erythrocyte sedimentation rate, and functional status in patients with AS or axial spondyloarthritis [11,12,13,14]. Recent studies have further strengthened interest in these parameters. Rapapa et al. reported higher NLR, MLR, SII, and SIRI values in patients with active AS [13], while Kaymaz-Tahra et al. identified PLR as an independent factor associated with active disease in a multivariable model [11]. Hematological inflammatory indices have also been investigated as potential indicators of response to pharmacological treatment, including tumor necrosis factor inhibitor therapy [15]. However, these indices are nonspecific and are influenced by several demographic, clinical, and lifestyle-related factors. They should therefore be considered complementary measures and not replacements for established clinical assessments and acute-phase reactants [10,16].

Whole-body cryotherapy (WBC) is a physical intervention involving short-term exposure of the entire body to extremely low temperatures, typically below −100 °C, under controlled conditions. The exposure to cryogenic temperatures induces thermoregulatory responses, including peripheral vasoconstriction, and may modify nociceptive transmission and neuromuscular function, thereby producing short-term analgesic effects and facilitating subsequent therapeutic exercise [17]. Previous studies in patients with AS have suggested that WBC combined with exercise therapy may reduce pain and disease activity and improve spinal mobility, physical function, and selected biochemical parameters [18,19,20,21,22]. Repeated WBC combined with kinesiotherapy has also been associated with attenuation of oxidative stress and changes in selected inflammatory and endothelial markers in patients with AS [19,20,23]. These findings suggest that analgesic, oxidative, inflammatory, and endothelial pathways may contribute to the observed clinical responses; however, their relative importance and causal role remain uncertain. Kinesiotherapy is a fundamental component of AS management and complements WBC through spinal and peripheral-joint mobilization, strengthening, respiratory exercises, and aerobic exercises intended to reduce stiffness and preserve or improve mobility, muscle function, chest expansion, and functional capacity [7,8,24]. A recent systematic review and meta-analysis reported significant improvements in the BASDAI, Ankylosing Spondylitis Disease Activity Score, Bath Ankylosing Spondylitis Functional Index, and VAS pain following WBC use as an adjunctive intervention [25]. However, the available studies were heterogeneous in terms of exposure protocols, treatment duration, and evaluated outcomes. Moreover, the pooled effect on CRP was not statistically significant, indicating that the biological mechanisms and systemic anti-inflammatory effects of WBC in AS remain incompletely understood [25].

Although complete blood count-derived inflammatory indices are increasingly being investigated as potential measures of disease activity and treatment response in AS, previous research has been predominantly cross-sectional or has focused on responses to pharmacological treatment [11,12,13,14,15]. Conversely, studies of WBC in AS have evaluated clinical outcomes, conventional inflammatory markers, cytokines, and oxidative stress, but they have not examined NLR, PLR, MLR, SII, and SIRI as a combined panel [18,19,20,21,22,23,24,25]. To the best of our knowledge, no previous study has examined these indices following WBC in men with AS. Because the specific CBC-derived indices and their associations with hsCRP, BASDAI, and VAS pain were not prespecified in the original study protocol, the present analyses were exploratory rather than hypothesis-driven. No directional or confirmatory hypothesis was formulated. The aim of this exploratory post hoc secondary analysis was to examine whether the addition of WBC to kinesiotherapy was associated with differences in NLR, PLR, MLR, SII, and SIRI compared with kinesiotherapy alone and to explore their associations with hsCRP, disease activity, and pain.

## 2. Materials and Methods

### 2.1. Study Design

This study is an exploratory post hoc secondary analysis of prospectively collected data from a parent study evaluating whole-body cryotherapy combined with kinesiotherapy in patients with ankylosing spondylitis. The original study design, eligibility criteria, allocation procedure, and intervention protocol have been described previously [20,23,24].

The previous publications arising from the parent study involved the same cohort of 32 men with AS, the same intervention groups (WBC combined with kinesiotherapy and kinesiotherapy alone), and the same intervention period and assessment time points as the present analysis [20,23,24]. These reports focused on oxidative-stress outcomes [20]; inflammatory, oxidative-stress, and endothelial parameters [23]; and clinical outcomes, including BASDAI, BASFI, spinal mobility, and VAS pain [24]. Accordingly, the previously reported hsCRP, BASDAI, and VAS pain outcomes are included in the present manuscript only as contextual variables for the exploratory association analyses and are not presented as novel findings.

Complete blood count parameters, hsCRP concentrations, BASDAI scores, and VAS pain scores were collected prospectively at the same study time points as part of the original study. The complete blood count data were included in the research plan but have not been reported in any previous publication. However, the specific evaluation of the derived inflammatory indices—NLR, PLR, MLR, SII, and SIRI—and their associations with hsCRP, BASDAI, and VAS pain were not explicitly prespecified in the original study protocol. These indices were therefore calculated post hoc from the original individual-level complete blood count data using established formulas. No additional blood sampling or laboratory measurements were performed.

### 2.2. Participants

Potential participants with ankylosing spondylitis (AS) were initially assessed by physicians at a rheumatology outpatient clinic. Only patients who met the predefined clinical eligibility criteria were referred to the study center for final confirmation of eligibility and possible enrolment. Consequently, the study investigators did not screen the entire outpatient clinic population and did not have access to the number of patients assessed or excluded at the initial referral stage.

Thirty-two patients were referred to the study center. Eligibility was confirmed by the study physician based on the predefined inclusion and exclusion criteria, a medical examination, and resting electrocardiography. All 32 referred patients met the eligibility criteria, provided written informed consent, and were enrolled. The final cohort comprised 32 men with AS, none of whom smoked or had previously undergone cryotherapy.

At baseline, the groups did not differ significantly in age, body mass index, disease activity—assessed using the Bath Ankylosing Spondylitis Disease Activity Index (BASDAI)—or pain intensity—assessed using a visual analogue scale (VAS). Selected baseline characteristics are presented in Table 1.

No formal a priori sample-size calculation was performed for the original study. Recruitment was limited by the availability of patients with AS who met the restrictive eligibility criteria. The present exploratory secondary analysis included all 32 participants with complete baseline and post-treatment CBC data. This study was not designed or powered specifically to evaluate treatment effects on the five CBC-derived indices or group-specific correlations with hsCRP, BASDAI, and VAS pain.

All participants met the modified New York criteria for definite AS [26]. Eligibility was additionally restricted to HLA-B27-positive patients with radiographic grade II or III sacroiliitis and clinically active disease. The same eligibility criteria were applied to both intervention groups.

Patients continued treatment with non-steroidal anti-inflammatory drugs (NSAIDs). The types and daily doses of NSAIDs used were comparable between the two treatment groups. Medication doses had remained stable for at least one month before enrolment and were not modified during the intervention period. No participant changed the type or dose of NSAID during this study. None of the participants received conventional synthetic disease-modifying antirheumatic drugs (csDMARDs), biological disease-modifying antirheumatic drugs (bDMARDs), or systemic glucocorticoids.

Patients were excluded if they had any contraindication to WBC or kinesiotherapy, concomitant diseases that could affect the assessed outcomes, or current tobacco use. Additional exclusion criteria comprised the use of vitamins, dietary supplements, hormonal preparations, immunomodulatory agents, or immunostimulatory products during the four weeks preceding the intervention. Previous exposure to any form of cryotherapy also precluded participation.

The participants were instructed to maintain their usual dietary habits throughout this study. Before blood sampling, they were asked to avoid caffeine-containing products for 12 h. No additional dietary intervention was introduced.

Before entering the intervention programme, each participant underwent a medical examination and resting electrocardiography to exclude potential contraindications to WBC. Blood pressure was measured before every cryotherapy session as part of the safety monitoring procedure.

The primary researcher assigned participants to the two intervention groups in an alternating 1:1 ratio, according to enrolment order.

### 2.3. Participant Allocation

The primary researcher allocated participants to the two intervention groups in an alternating 1:1 sequence according to the order of enrolment. The WBC + K group included 16 patients who received whole-body cryotherapy followed by kinesiotherapy, whereas the KT group comprised 16 patients who participated in the same kinesiotherapy programme without exposure to WBC. Owing to the nature of the intervention, participants and physiotherapists delivering the treatment could not be blinded to group allocation. BASDAI and VAS pain were patient-reported outcomes and were therefore assessed by participants who were aware of their treatment allocation.

**Figure 1:**
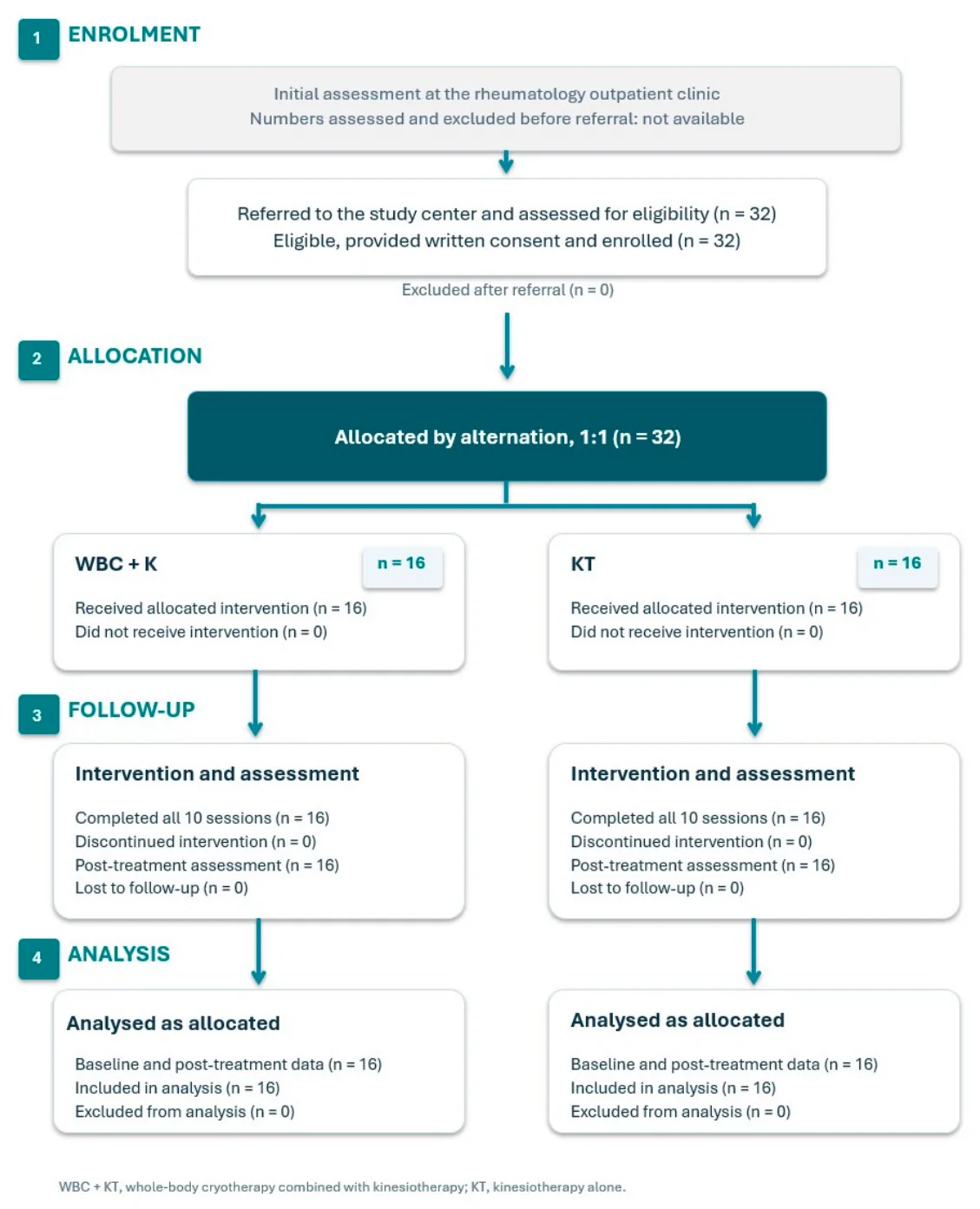
Participant flow throughout this study. The investigators did not have access to the number of patients screened or excluded at the initial rheumatology outpatient clinic referral stage. WBC + K, whole-body cryotherapy combined with kinesiotherapy; KT, kinesiotherapy alone.

All participants completed all ten scheduled treatment sessions and underwent the post-intervention assessment. There were no withdrawals, losses to follow-up, or exclusions. Complete baseline and post-intervention data were available for all 32 participants, and all participants were included in the present secondary analysis. No adverse events or complications related to WBC or kinesiotherapy were observed. Participant flow is presented in Figure 1.

### 2.4. Whole-Body Cryotherapy Protocol

The WBC and kinesiotherapy protocols were based on procedures described in our previous studies [20,23,24]. Participants assigned to the WBC+K group completed 10 sessions in a liquid-nitrogen-cooled, Wrocławski-type cryochamber (Creator, Wroclaw, Poland). The treatment was administered on five consecutive weekdays per week over a two-week period, with all sessions conducted in the morning.

Each session began with a 30 s acclimatization period in the antechamber at −60 °C. Participants subsequently entered the main chamber, where they remained at approximately −120 °C for 3 min. During cold exposure, they walked slowly around the chamber and reversed their walking direction every 30 s. They were instructed to maintain slow, regular breathing throughout the procedure.

The oxygen concentration inside the chamber was continuously monitored and maintained at 21–22%. Every session was supervised by trained personnel, and the procedure could be discontinued immediately at the request of either the participant or the supervisor.

During cryotherapy, participants wore shorts, woollen knee-length socks, gloves, clogs, and a headband or cap covering the auricles. Their nose and mouth were protected with a surgical mask. Before entering the chamber, participants were required to dry their skin thoroughly. Jewellery, watches, contact lenses, and other items unsuitable for exposure to extremely low temperatures were removed when applicable.

### 2.5. Kinesiotherapy Programme

Immediately after each WBC session, participants in the WBC + K group commenced a standardized 60 min kinesiotherapy programme. All WBC procedures were performed between 10:00 and 11:00 a.m. Participants in the KT group performed the same kinesiotherapy programme within the corresponding morning time window but without prior exposure to WBC. In both groups, the programme was conducted on five consecutive weekdays per week over a two-week period, for a total of ten sessions.

Each 60 min session comprised approximately 25 min of mobility, respiratory, and strengthening exercises; 15 min of stationary cycling; and 20 min of brisk walking. Mobility exercises were intended to maintain or improve the range of motion of the cervical, thoracic, and lumbar spine and the major peripheral joints, including the shoulders, elbows, wrists, hips, knees, and ankles. Respiratory exercises were intended to improve chest expansion and respiratory function. Strengthening exercises targeted the principal muscle groups of the trunk, upper limbs, and thighs. Each mobility, respiratory, and strengthening exercise was performed for 10 repetitions.

All sessions were conducted under the direct supervision of qualified physiotherapists to ensure correct exercise performance and participant safety. The physiotherapists monitored attendance, exercise tolerance, and the participants’ clinical condition throughout each session. The structure, duration, and types of exercises were standardized across both groups, whereas exercise intensity was individually adjusted according to each participant’s functional capacity, pain, and tolerance. Intensity was monitored clinically by the supervising physiotherapist; no predefined heart-rate or rating-of-perceived-exertion target was used.

### 2.6. Biochemical Analysis and Calculation of Complete Blood Count Indices

Blood samples were collected at two time points: in the morning on the day preceding the first intervention and in the morning on the day after completion of the ten-session treatment programme. At both time points, venous blood was obtained after an overnight fast and before the participants’ first meal. Post-treatment blood collection was performed approximately 24 h after the final intervention session according to the same schedule in both groups.

Blood was drawn from a forearm vein using the S-Monovette system (Sarstedt, Nümbrecht, Germany). Samples used for complete blood count measurements were collected into 2.7 mL tubes containing K3-EDTA at a concentration of 1.6 mg/mL. Immediately after collection, the tubes were gently inverted to ensure adequate mixing with the anticoagulant, and they were maintained at room temperature until analysis. The samples were neither centrifuged nor frozen and were analyzed in whole blood promptly after collection, in accordance with the laboratory’s standard operating procedures.

Complete blood count parameters were measured using an ABX PENTRA 60 hematology analyzer (ABX Diagnostics, Montpellier, France). Internal quality control was performed daily, before the analysis of participant samples, using commercially available ABX Minotrol 16 whole-blood hematology control material. Participant samples were analyzed only after the quality-control results had been confirmed to be within the manufacturer-defined acceptance ranges. The analyzer was calibrated in accordance with the manufacturer’s instructions and the laboratory’s standard operating procedures.

Samples were identified using coded participant identifiers, and laboratory personnel did not have access to the treatment-allocation list during the analyses.

Samples intended for hsCRP measurement were collected separately into 2.7 mL tubes containing a clot activator. After complete clot formation at room temperature, the samples were centrifuged at 900× *g* for 10 min at 4 °C. The resulting serum was aliquoted immediately and stored at −75 °C until analysis. Serum hsCRP concentrations were determined using a latex-enhanced immunoturbidimetric assay (BioSystems, Barcelona, Spain). The intra-assay and inter-assay coefficients of variation were 2.4% and 5.6%, respectively.

The CBC-derived inflammatory indices were calculated using the following formulas: NLR = neutrophil count/lymphocyte count; PLR = platelet count/lymphocyte count; MLR = monocyte count/lymphocyte count; SII = (platelet count × neutrophil count)/lymphocyte count; and SIRI = (neutrophil count × monocyte count)/lymphocyte count [12,27].

### 2.7. Clinical State Estimation

Pain intensity was assessed using a 10 cm visual analogue scale (VAS), ranging from 0 (no pain) to 10 (worst imaginable pain). The assessment was performed at baseline and after completion of the intervention programme. Higher VAS scores indicated greater pain intensity.

Disease activity was assessed using the Bath Ankylosing Spondylitis Disease Activity Index (BASDAI). The questionnaire comprises six items evaluating fatigue, spinal pain, peripheral joint pain and swelling, localized tenderness suggestive of enthesitis, and the severity and duration of morning stiffness. Each item is rated on a scale from 0, indicating no symptoms, to 10, indicating the greatest symptom severity. The BASDAI score was calculated by first determining the mean of the two morning stiffness items, adding this value to the scores for the remaining four items, and dividing the resulting sum by five. The final score ranges from 0 to 10, with higher values indicating greater disease activity [28].

### 2.8. Statistical Analysis

Statistical analyses were performed using Statistica version 13.3 PL (TIBCO Software Inc., Palo Alto, CA, USA) and Python version 3.12.14 (Python Software Foundation, Wilmington, DE, USA), with pandas version 2.2.3, NumPy version 2.3.5, and SciPy version 1.17.0. Baseline-adjusted ANCOVA models with HC3 heteroscedasticity-consistent standard errors, adjusted marginal means, 95% confidence intervals, effect-size estimates, bootstrap analyses, log-transformed sensitivity analyses, and Benjamini–Hochberg corrections were performed in Python.

Data distributions were assessed using the Shapiro–Wilk test, graphical inspection, and evaluation of extreme observations. Parametrically analyzed variables are presented as mean ± standard deviation (SD), non-parametrically analyzed variables are presented as median [interquartile range, IQR], and categorical variables are presented as numbers and percentages. All 32 participants had complete baseline and post-treatment data; therefore, no imputation was required.

The primary intervention effects on NLR, PLR, MLR, SII, and SIRI were assessed using separate baseline-adjusted ANCOVA models, with the post-treatment value as the dependent variable, treatment group as the fixed factor, and the corresponding baseline value as a covariate. Adjusted marginal means and adjusted between-group differences, calculated as WBC + K minus KT, are reported with 95% CIs. HC3 standard errors were used for statistical inference. Linearity, homogeneity of regression slopes, residual distributions, Cook’s distance, and leverage were examined. Robustness was evaluated using stratified bootstrap percentile 95% CIs based on 10,000 resamples and ANCOVA of natural-log-transformed values. Effect sizes were expressed as partial eta squared (partial η^2^), calculated from the corresponding conventional ANCOVA models. The Benjamini–Hochberg procedure was applied across the five baseline-adjusted treatment effects.

Change scores were calculated as Δ = post-treatment value − baseline value and analyzed as a sensitivity analysis using Welch’s independent-samples *t*-test or the Mann–Whitney U test, as appropriate. Effects were reported as mean differences with 95% CIs and Hedges’ *g* for parametric comparisons or as Hodges–Lehmann estimates with distribution-free 95% CIs and rank-biserial correlations for non-parametric comparisons. The five change-score comparisons of the CBC-derived indices constituted a separate family for Benjamini–Hochberg correction. Baseline and post-treatment between-group comparisons were performed using Welch’s *t*-test or the Mann–Whitney U test. Within-group changes were assessed using the paired-samples *t*-test or Wilcoxon signed-rank test, with Hedges’ g_av or matched-pairs rank-biserial correlations reported as the corresponding effect sizes. These unadjusted comparisons were considered supportive or descriptive.

Associations between the CBC-derived indices and hsCRP, BASDAI, and VAS pain were assessed separately in each group using Spearman’s rank correlation coefficient. Bootstrap percentile 95% CIs were calculated using 10,000 within-group resamples. The Benjamini–Hochberg procedure was applied separately to each family of 15 change-to-change correlations and 15 post-treatment correlations within each treatment group. Correlation coefficients were not formally compared between groups.

All tests were two-sided. Unadjusted probabilities are reported as *p*-values, and Benjamini–Hochberg-adjusted probabilities are reported as *q*-values. Values are reported to three decimal places, with values below 0.001 presented as *p* < 0.001 or *q* < 0.001. Statistical significance was defined as *p* < 0.05 or *q* < 0.05, as appropriate. No formal a priori sample-size calculation was performed for the original study or the present post hoc secondary analysis.

## 3. Results

The results of the primary baseline-adjusted ANCOVA, including the corresponding effect sizes expressed as partial η^2^, are presented in Table 2. None of the adjusted between-group effects were statistically significant either before or after correction for multiple comparisons. Unadjusted pre–post values and change-score sensitivity analyses for the CBC-derived indices are presented in Table 3, whereas the corresponding analyses of the previously reported contextual outcomes are presented in Supplementary Table S1.

### 3.1. Complete Blood Count-Derived Inflammatory Indices and hsCRP

Baseline MLR and SIRI values were higher in the WBC + K group than in the KT group. Mean MLR was 0.242 ± 0.051 versus 0.188 ± 0.055, and median SIRI was 0.962 [0.753–1.379] versus 0.667 [0.489–1.061] ×10^9^/L, respectively (Table 3). The observed baseline imbalances were accounted for in the primary analysis by incorporating the corresponding baseline values as covariates.

The primary assessment of the intervention effect was based on baseline-adjusted analysis of covariance (ANCOVA), with the post-treatment value as the dependent variable, treatment group as the fixed factor, and the corresponding baseline value as a covariate. None of the adjusted between-group treatment effects for NLR, PLR, MLR, SII, or SIRI were statistically significant, either before or after Benjamini–Hochberg correction. Thus, the primary analysis did not provide multiplicity-adjusted evidence of an additional effect of WBC on any of the CBC-derived inflammatory indices. The corresponding effect sizes, expressed as partial η^2^, are presented in Table 2.

Among the five baseline-adjusted comparisons, MLR yielded the lowest unadjusted *p*-value. The adjusted post-treatment mean was 0.177 (95% CI: 0.151 to 0.202) in the WBC + K group and 0.209 (95% CI: 0.176 to 0.243) in the KT group, corresponding to an adjusted between-group difference of −0.033 (95% CI: −0.074 to 0.008; *p* = 0.115; *q* = 0.329; partial η^2^ = 0.066). Thus, the adjusted effect was not statistically significant; its 95% confidence interval included zero, and the result remained non-significant after Benjamini–Hochberg correction. The adjusted differences for NLR, PLR, SII, and SIRI were also not statistically significant, and all corresponding 95% confidence intervals included zero. No statistically significant group-by-baseline interactions were identified, providing no evidence against the homogeneity-of-regression-slopes assumption (Table 2).

In the unadjusted change-score sensitivity analysis, the reduction in MLR was greater in the WBC + K group than in the KT group (−0.051 ± 0.053 vs. 0.007 ± 0.069), with a large standardized effect (Hedges’ *g* = −0.927). This difference was nominally significant (*p* = 0.012) but did not remain statistically significant after Benjamini–Hochberg correction across the five CBC-derived indices (*q* = 0.060). Differences in changes in NLR, PLR, SII, and SIRI were not statistically significant even before multiplicity adjustment (Table 3).

In the descriptive within-group analyses, PLR, MLR, and SIRI decreased significantly in the WBC + K group (*p* = 0.008, *p* = 0.002, and *p* = 0.034, respectively), whereas no significant changes in these indices were observed in the KT group. These within-group findings were considered descriptive and were not interpreted as evidence of a differential treatment effect (Table 3).

In the descriptive analysis of individual hematological parameters, the lymphocyte count increased significantly in the WBC + K group (*p* < 0.001), whereas no significant change was observed in the KT group (*p* = 0.945). However, the between-group difference in lymphocyte-count change was not statistically significant (*p* = 0.174). No significant between-group differences were observed for changes in total leukocyte, platelet, neutrophil, or monocyte counts (Table 3).

For hsCRP, which was included as a previously reported contextual inflammatory variable, a significant reduction was observed in the WBC + K group, from 9.350 [2.270–15.930] mg/L at baseline to 2.750 [0.750–11.380] mg/L after treatment (*p* = 0.002). No significant change was observed in the KT group, in which the corresponding values were 9.000 [3.300–15.350] and 7.00 [3.020–17.680] mg/L (*p* = 0.632). The reduction in hsCRP was greater in the WBC + K group than in the KT group (median change: −3.650 [−5.850 to −0.800] vs. 0.400 [−0.870 to 2.120] mg/L; *p* = 0.006), whereas post-treatment hsCRP concentrations did not differ significantly between the groups (*p* = 0.175). Effect sizes for the within-group and between-group changes in hsCRP, BASDAI, and VAS pain are presented in Supplementary Table S1.

### 3.2. Disease Activity and Pain

BASDAI scores decreased significantly in both groups (both *p* < 0.001). Mean BASDAI scores decreased from 5.430 ± 1.610 to 3.290 ± 0.910 points in the WBC + K group and from 5.280 ± 1.710 to 4.530 ± 1.620 points in the KT group. The reduction was greater in the WBC + K group than in the KT group (median change: −1.900 [−2.950 to −1.470] vs. −0.600 [−0.820 to −0.570] points; *p* < 0.001)**,** with a between-group rank-biserial correlation of *r* rb = −0.707. Post-treatment BASDAI scores were significantly lower in the WBC + K group than in the KT group (*p* = 0.014; Supplementary Table S1).

AS pain scores decreased significantly in both the WBC + K and KT groups (*p* < 0.001 and *p* = 0.001, respectively). At baseline, mean VAS pain scores were 5.250 ± 1.730 points in the WBC + K group and 5.000 ± 1.630 points in the KT group. After treatment, the corresponding median values were 3.000 [1.380–4.000] and 4.000 [2.750–5.000] points, respectively. The reduction was greater in the WBC + K group than in the KT group (median change: −1.500 [−2.250 to −1.000] vs. −1.000 [−1.000 to −0.750] points; *p* = 0.021), with a between-group rank-biserial correlation of *r*_rb = −0.449. However, post-treatment VAS pain scores did not differ significantly between the groups (*p* = 0.072; Supplementary Table S1).

### 3.3. Correlation Analysis

Complete results of the change-to-change correlation analyses are presented in Table 4. Fifteen associations between changes in the five CBC-derived inflammatory indices and changes in hsCRP, BASDAI, and VAS pain were examined separately in each treatment group. None of the correlations remained statistically significant after Benjamini–Hochberg correction in either the WBC + K or KT group. The bootstrap 95% confidence intervals were generally wide and frequently included both negative and positive values, indicating substantial uncertainty in the correlation estimates. Accordingly, these analyses did not provide evidence that changes in the CBC-derived inflammatory indices were associated with changes in hsCRP, disease activity, or pain (Table 4).

Complete results of the exploratory post-treatment correlation analyses are provided in Supplementary Table S2. Several nominal associations were observed in the WBC + K group: BASDAI with MLR (*r*_s_ = 0.566, 95% CI: 0.165 to 0.790, *p* = 0.022, *q* = 0.154), BASDAI with SIRI (*r*_s_ = 0.499, 95% CI: −0.021 to 0.853, *p* = 0.049, *q* = 0.154), VAS pain with NLR (*r*_s_ = 0.620, 95% CI: 0.156 to 0.926, *p* = 0.010, *q* = 0.154), and VAS pain with SII (*r*_s_ = 0.498, 95% CI: 0.065 to 0.785, *p* = 0.050, *q* = 0.154). In the KT group, a nominal inverse association was observed between hsCRP and MLR (*r*_s_ = −0.549, 95% CI: −0.848 to −0.063, *p* = 0.028, *q* = 0.413). However, none of the post-treatment correlations remained statistically significant after correction for multiple testing.

## 4. Discussion

To the best of our knowledge, this is the first study to investigate whether adding WBC to kinesiotherapy was associated with differences in NLR, PLR, MLR, SII, and SIRI in men with AS. In the primary baseline-adjusted ANCOVA, none of the adjusted between-group treatment effects for the five CBC-derived inflammatory indices were statistically significant before or after correction for multiple testing. Therefore, the present exploratory post hoc secondary analysis did not provide evidence of an additional effect of WBC on any of the investigated indices. The strongest adjusted effect was observed for MLR; however, the adjusted between-group difference was not statistically significant, and its 95% confidence interval included zero. Although the change-score sensitivity analysis indicated a nominally greater reduction in MLR in the WBC + K group, this signal did not meet the multiplicity-adjusted significance criterion. Similarly, the significant within-group reductions in PLR, MLR, and SIRI were descriptive and should not be interpreted as evidence of an additional or differential effect of WBC.

Complete blood count-derived inflammatory indices have increasingly been investigated as accessible measures of systemic inflammatory and immune activity. NLR, PLR, and MLR reflect the relative contributions of neutrophils, platelets, monocytes, and lymphocytes, whereas SII and SIRI integrate several cellular components into composite indices. Previous studies have reported higher values of selected indices in patients with AS or axial spondyloarthritis (axSpA) than in healthy individuals and associations with conventional inflammatory markers or clinical measures of disease activity [12,14,29,30,31,32]. NLR and PLR have been associated with BASDAI and acute-phase reactants, although the strength and consistency of these relationships have varied across studies [14,29,30]. SII has also been proposed as a potential indicator of disease activity in AS [12,31], while indices incorporating monocytes, including MLR and SIRI, may provide additional information about immune-inflammatory activity [12,32]. However, most previous studies have focused on cross-sectional associations or responses to pharmacological treatment, whereas WBC studies in AS have primarily evaluated clinical outcomes, conventional inflammatory markers, cytokines, and oxidative stress [18,19,20,21]. The present analysis connects these two areas of research but, given the null baseline-adjusted findings, does not establish that CBC-derived indices are specifically responsive to the addition of WBC.

The interpretation of MLR and SIRI requires particular consideration of their baseline imbalance. Both indices were significantly higher at baseline in the WBC + K group, making analyses based exclusively on change scores potentially sensitive to regression to the mean. Accordingly, baseline-adjusted ANCOVA was used as the primary analysis, and neither MLR nor SIRI showed a statistically significant adjusted treatment effect. The nominal MLR change-score result should therefore be regarded only as a sensitivity-analysis signal. Although MLR decreased within the WBC + K group, this finding should not be regarded as a confirmatory intervention effect. Because MLR is calculated by dividing the monocyte count by the lymphocyte count, its decrease was arithmetically consistent primarily with the observed increase in lymphocyte count, whereas the monocyte count did not change significantly. Nevertheless, the between-group difference in lymphocyte-count change was not statistically significant. Thus, the present findings neither establish that the MLR change was caused by WBC nor support a specific immunological mechanism.

Although PLR and SIRI decreased in the within-group analyses, neither their baseline-adjusted treatment effects nor their between-group change-score comparisons were statistically significant. Neither NLR nor SII changed significantly. The heterogeneous behavior of the indices may reflect differences in their cellular components, baseline values, biological variability, the small sample size, or the short intervention period. Accordingly, these indices should not be considered interchangeable measures of inflammation, and isolated within-group changes should not be interpreted as treatment-specific effects.

The previously reported hsCRP, BASDAI, and VAS pain outcomes were included as contextual variables. The unadjusted reduction in hsCRP was greater in the WBC + K group than after kinesiotherapy alone. However, hsCRP was not included in the multiplicity family defined for the five CBC-derived indices, and this finding does not alter the primary null result. Moreover, neither hsCRP nor the CBC-derived indices can, by themselves, establish a generalized systemic anti-inflammatory mechanism. Previous studies of WBC in AS have not consistently demonstrated changes in conventional inflammatory markers. Improvements in disease activity, function, pain, oxidative stress, and selected cardiovascular risk-related parameters have been reported, whereas changes in CRP were not consistently observed [18,19]. Differences in baseline inflammatory activity, exposure protocols, cryochamber conditions, concomitant treatment, sample size, and assay characteristics may partly explain these discrepancies.

Both groups experienced significant improvements in BASDAI and VAS pain, with greater unadjusted reductions in the WBC + K group. In the WBC + K group, mean BASDAI decreased from 5.43 to 3.29 and therefore fell below the commonly used threshold of 4 for active disease [33]. This represents a clinically relevant improvement in self-reported disease activity at the group level, but it does not indicate that every participant achieved inactive disease or remission. BASDAI is a continuous patient-reported measure, whereas formal disease activity states are more appropriately classified using ASDAS; interpretation based on a group mean also does not reflect individual variability in response [7,33].

Recent evidence supports exercise therapy as an effective non-pharmacological component of axSpA management. A systematic review and meta-analysis reported improvements in disease activity, physical function, spinal mobility, and symptom control following structured exercise while also identifying heterogeneity in exercise protocols and limitations in the quality of evidence [34]. The clinical relevance of sustained exercise therapy has also been supported by a one-year study in patients with axSpA and severe functional limitations [35]. These findings are important because kinesiotherapy was administered in both groups and likely contributed to the improvements in BASDAI and pain. Consequently, improvement within the WBC + K group cannot be attributed specifically to WBC.

In addition to the established benefits of exercise therapy, previous studies have suggested that WBC used as an adjunct to rehabilitation may further improve functional outcomes in patients with AS [18,24]. Exposure to extremely low temperatures may affect pain perception through reduced nerve conduction velocity, modulation of nociceptive signaling, and changes in inflammatory or neuroendocrine mediators [36,37]. WBC may also facilitate participation in therapeutic exercise by temporarily reducing pain and muscle tension [17,18]. Nevertheless, the present study cannot distinguish direct biological effects of cold exposure from indirect effects mediated through improved tolerance of, or participation in, subsequent exercise. This further supports interpreting WBC as a potential adjunct rather than attributing the observed clinical improvement exclusively to cold exposure.

Previous research indicates that WBC may affect circulating leukocyte populations, although the direction and magnitude of these responses depend on the exposure protocol and number of sessions [38,39]. However, CBC-derived indices do not identify lymphocyte or monocyte subpopulations and cannot distinguish changes in cell production, redistribution, activation, function, or survival. The present study was not designed to investigate these mechanisms, and the observed laboratory changes may reflect transient redistribution, biological variability, exercise responses, cold exposure, or their combination. Future studies incorporating repeated blood sampling, immunophenotyping, functional cellular assays, and cytokine measurements are required to clarify the biological basis and duration of any such responses.

The primary correlation analysis did not demonstrate significant associations between changes in hsCRP, BASDAI, or VAS pain and changes in NLR, PLR, MLR, SII, or SIRI in either treatment group. Several nominal post-treatment associations were identified, but none remained statistically significant after correction for multiple testing. Because the post-treatment analyses were cross-sectional, involved only 16 participants per group, and did not formally compare correlation coefficients between groups, they should be regarded strictly as exploratory. The present findings therefore do not demonstrate that the investigated indices track systemic inflammation, disease activity, pain severity, or treatment response. Larger prospective studies with prespecified hypotheses, repeated measurements, and sufficient power to compare associations between groups are required before any monitoring role can be proposed.

From a clinical perspective, CBC-derived inflammatory indices are inexpensive, readily calculated from routine laboratory results, and integrate information from several circulating cell populations [11,12,13,14,31,32]. However, they are nonspecific and may be influenced by demographic characteristics, smoking, body mass index, physical activity, comorbidities, medication, infection, stress, and other factors [40]. Their potential role in AS should therefore be regarded as complementary to established assessments such as hsCRP, BASDAI, ASDAS, and clinical examination rather than as a replacement for them.

Several limitations should be acknowledged. The present study is an exploratory post hoc secondary analysis. Although CBC parameters were collected prospectively together with hsCRP, BASDAI, and VAS pain, the specific CBC-derived indices and association analyses were not explicitly prespecified in the original protocol. The parent study was not designed or powered specifically to evaluate between-group effects on these indices. With only 16 participants per group, treatment-effect estimates were imprecise, and the group-specific correlation analyses produced wide bootstrap confidence intervals. Baseline differences in MLR and SIRI further made the unadjusted change-score analyses susceptible to regression to the mean, although this concern was addressed using baseline-adjusted ANCOVA as the primary analysis. The change-score comparisons were retained only as sensitivity analyses, and the within-group analyses were interpreted descriptively.

The use of alternating allocation rather than true randomization rendered treatment assignment potentially predictable and may have introduced selection bias. The short intervention period and absence of longer-term follow-up also preclude conclusions regarding the persistence of the observed changes. Blood was sampled only before the first intervention and on the morning after completion of the ten-session programme; no intermediate or subsequent follow-up samples were obtained. Consequently, the temporal pattern of the laboratory findings could not be assessed, and transient physiological responses to repeated cold exposure, exercise, or their combination cannot be distinguished from sustained biological effects.

This study did not include immune-cell phenotyping, functional cellular assays, or an extensive panel of inflammatory mediators. Because the CBC-derived indices and hsCRP are nonspecific surrogate measures, they cannot establish a specific cellular, immunological, or generalized anti-inflammatory mechanism. ASDAS-CRP was also unavailable because the parent study did not collect all patient-reported components required for its valid calculation, limiting comparisons with the currently preferred measure of axSpA disease activity.

The study population was highly homogeneous and exclusively included HLA-B27-positive, non-smoking men with active radiographic AS and grade II–III sacroiliitis who were receiving stable NSAID therapy but no biological or targeted synthetic disease-modifying antirheumatic drugs. Although this homogeneity reduced several sources of confounding, it substantially limits generalizability. The cohort does not reflect the broader contemporary axSpA population, which includes women, patients with non-radiographic axSpA, individuals with different HLA-B27 status, smokers, patients with relevant comorbidities, and those receiving biological or targeted synthetic therapies. Robust population-based data on the prevalence and demographic characteristics of AS in Poland are also limited, restricting direct assessment of the cohort’s national representativeness. The findings should therefore not be extrapolated beyond this narrowly defined population.

Despite these limitations, this study provides preliminary data on CBC-derived inflammatory indices following WBC combined with kinesiotherapy. The primary baseline-adjusted analysis did not demonstrate an additional effect of WBC on NLR, PLR, MLR, SII, or SIRI. The nominal MLR signal and the effect-size estimates for selected outcomes may justify further investigation, but they should be regarded as exploratory rather than confirmatory. Larger randomized controlled studies should prospectively prespecify the outcomes and analysis plan; use concealed random allocation and adequate statistical power; standardize WBC and exercise protocols; incorporate repeated blood sampling and longer follow-up; and include detailed immunological assessments. Such studies are needed to determine whether WBC has an additional effect on CBC-derived inflammatory indices and whether any observed changes have clinical relevance.

## 5. Conclusions

This exploratory post hoc secondary analysis did not provide evidence that adding whole-body cryotherapy to kinesiotherapy had an additional effect on NLR, PLR, MLR, SII, or SIRI in the studied cohort of HLA-B27-positive men with active radiographic AS receiving NSAID therapy. None of the treatment effects estimated using baseline-adjusted ANCOVA were statistically significant before or after correction for multiple testing. The nominal between-group difference in MLR change identified in the change-score sensitivity analysis did not remain statistically significant after Benjamini–Hochberg corrections. Although PLR, MLR, and SIRI decreased in the within-group analyses of patients receiving WBC combined with kinesiotherapy, these findings do not establish an additional effect of WBC and should be considered descriptive and hypothesis-generating. Changes in the CBC-derived inflammatory indices were not significantly associated with changes in hsCRP, disease activity, or pain. Larger, prospectively designed, adequately powered randomized controlled trials with prespecified outcomes and baseline-adjusted analyses are required to determine whether WBC has an additional effect on these indices.

## Figures and Tables

**Table 1:** Pre-treatment demographic and clinical profile of participants according to treatment allocation. The study population and intervention protocol were described previously [20,23,24].

Characteristic	WBC + K Group (*n* = 16)	KT Group (*n* = 16)	*p*-Value
Age, years	45.940 ± 1.240	46.630 ± 1.500	0.114
Sex, male/female, *n*	16/0	16/0	—
BMI, kg/m^2^	24.240 ± 4.400	23.760 ± 6.800	0.880
hsCRP, mg/L	9.350 [2.270–15.930]	9.000 [3.300–15.350]	0.806
BASDAI, points	5.430 ± 1.610	5.280 ± 1.710	0.792
VAS pain, points	5.250 ± 1.730	5.000 ± 1.630	0.676
Current smoking, yes/no, *n*	0/16	0/16	—
Medication, yes/no, *n*			
NSAIDs	16/0	16/0	—
csDMARDs	0/16	0/16	—
bDMARDs	0/16	0/16	—
Systemic glucocorticoids	0/16	0/16	—

Note: Continuous data are presented as mean ± standard deviation (SD) for variables summarized parametrically and as median [interquartile range, IQR] for variables summarized non-parametrically. Categorical data are presented as numbers of participants. The *p*-values refer to between-group comparisons at baseline. hsCRP was compared using the Mann–Whitney U test; the remaining continuous variables were compared using Welch’s independent-samples *t*-test. The *p*-value was not calculated for variables without variability. BMI, body mass index; hsCRP, high-sensitivity C-reactive protein; BASDAI, Bath Ankylosing Spondylitis Disease Activity Index; VAS, visual analogue scale; WBC + K, whole-body cryotherapy combined with kinesiotherapy; KT, kinesiotherapy alone; NSAIDs, non-steroidal anti-inflammatory drugs; csDMARDs, conventional synthetic disease-modifying antirheumatic drugs; bDMARDs, biological disease-modifying antirheumatic drugs.

**Table 2:** Primary baseline-adjusted ANCOVA and bootstrap sensitivity results for CBC-derived inflammatory indices.

Index	Adjusted Mean WBC + K (95% CI)	Adjusted Mean KT (95% CI)	Adjusted Between-Group Difference (95% CI)	Bootstrap 95% CI	*p*-Value	*q*-Value	Partial η^2^
NLR	2.155 (1.713–2.597)	2.448 (1.912–2.983)	−0.293 (−0.977–0.392)	−0.915–0.273	0.389	0.512	0.028
PLR	131.318 (111.587–151.048)	154.320 (131.796–176.843)	−23.002 (−53.316–7.313)	−48.535–3.773	0.132	0.329	0.084
MLR	0.177 (0.151–0.202)	0.209 (0.176–0.243)	−0.033 (−0.074–0.008)	−0.072–0.004	0.115	0.329	0.066
SII	588.113 (460.718–715.507)	658.645 (541.147–776.143)	−70.532 (−242.893–101.828)	−205.348–85.533	0.409	0.512	0.027
SIRI	0.832 (0.651–1.013)	0.846 (0.710–0.981)	−0.013 (−0.240–0.214)	−0.212–0.188	0.905	0.905	<0.001

ANCOVA, analysis of covariance; CI, confidence interval; HC3, heteroscedasticity-consistent covariance estimator type 3; KT, kinesiotherapy alone; MLR, monocyte-to-lymphocyte ratio; NLR, neutrophil-to-lymphocyte ratio; PLR, platelet-to-lymphocyte ratio; SII, systemic immune-inflammation index; SIRI, systemic inflammation response index; WBC + K, whole-body cryotherapy combined with kinesiotherapy. Adjusted marginal means were estimated at the overall mean of the corresponding baseline value. Adjusted differences were calculated as WBC + K minus KT. HC3 95% CIs and two-sided unadjusted *p*-values were obtained from the baseline-adjusted ANCOVA models. Bootstrap intervals are percentile bootstrap 95% CIs based on 10,000 within-group resamples. The *q*-values were calculated by applying the Benjamini–Hochberg procedure to the family of five baseline-adjusted treatment effects. The predefined inferential criterion was *q* < 0.05. Partial η^2^ was calculated from the corresponding conventional ANCOVA model.

**Table 3:** Unadjusted descriptive and change-score sensitivity analyses of complete blood count parameters and CBC-derived inflammatory indices.

Parameter	Assessment	WBC + K Group	KT Group	Between-Group Difference in Δ (95% CI)	Effect Size	Statistical Test	*p*-Value	*q*-Value
Component complete blood count parameters—supportive descriptive analyses								
Total leukocyte count (×10^9^/L)	Before	6.80 [5.83–7.40]	6.25 [5.25–7.72]	—	—	MWU	0.584	—
	After	6.75 [6.28–7.33]	6.40 [5.17–7.83]	—	—	MWU	0.300	—
	Within-group *p*	0.593	0.769	—	—	WBC + K: WSR; KT: PT	—	—
	Δ	0.00 [−0.43–0.55]	0.00 [−0.79–0.85]	0.20 (−0.60 to 1.06)	—	MWU	0.706	—
Platelet count (×10^9^/L)	Before	228.50 [202.25–255.00]	264.50 [237.50–349.50]	—	—	MWU	0.065	—
	After	259.75 ± 65.84	285.81 ± 61.23	—	—	WT	0.255	—
	Within-group *p*	0.098	0.983	—	—	WBC + K: WSR; KT: PT	—	—
	Δ	13.50 [−0.75–20.25]	13.00 [−22.50–27.25]	2.50 (−21.00 to 37.00)	—	MWU	0.851	—
Neutrophil count (×10^9^/L)	Before	4.259 [3.689–4.764]	4.098 [3.453–4.655]	—	—	MWU	0.534	—
	After	4.520 ± 1.264	4.194 ± 1.175	—	—	WT	0.455	—
	Within-group *p*	0.495	0.771	—	—	WBC + K: WSR; KT: PT	—	—
	Δ	−0.158 [−0.863–0.365]	−0.003 [−0.763–0.741]	−0.071 (−0.801 to 0.706)	—	MWU	0.865	—
Lymphocyte count (×10^9^/L)	Before	1.844 [1.709–2.034]	1.796 [1.371–2.310]	—	—	MWU	0.777	—
	After	2.243 ± 0.570	2.061 ± 0.990	—	—	WT	0.528	—
	Within-group *p*	<0.001	0.945	—	—	WBC + K: PT; KT: PT	—	—
	Δ	0.369 ± 0.316	−0.018 ± 1.048	0.388 (−0.188 to 0.963)	—	WT	0.174	—
Monocyte count (×10^9^/L)	Before	0.444 ± 0.103	0.365 ± 0.147	—	—	WT	0.093	—
	After	0.372 [0.341–0.496]	0.372 [0.252–0.448]	—	—	MWU	0.356	—
	Within-group *p*	0.149	0.831	—	—	WBC + K: PT; KT: PT	—	—
	Δ	−0.034 ± 0.090	−0.008 ± 0.143	−0.026 (−0.113 to 0.060)	—	WT	0.536	—
CBC-derived inflammatory indices—change-score sensitivity analyses								
NLR	Before	2.394 [1.843–2.837]	2.424 [1.681–2.915]	—	—	MWU	0.865	—
	After	1.997 [1.671–2.428]	2.309 [1.619–2.768]	—	—	MWU	0.559	—
	Within-group *p*	0.086	0.924	—	WBC + K: g_av = −0.369; KT: g_av = −0.025	WBC + K: PT; KT: PT	—	—
	Δ	−0.399 ± 0.870	−0.029 ± 1.189	−0.370 (−1.125 to 0.385)	g = −0.347	WT	0.323	0.356
PLR	Before	128.368 [105.230–153.135]	165.429 [118.991–194.158]	—	—	MWU	0.109	—
	After	115.234 [89.812–130.363]	164.730 [118.260–198.981]	—	—	MWU	0.062	—
	Within-group *p*	0.008	0.930	—	WBC + K: r_rb = −0.735; KT: g_av = 0.017	WBC + K: WSR; KT: PT	—	—
	Δ	−13.711 [−27.347 to −4.551]	13.921 [−14.235–28.966]	−23.723 (−47.293 to 4.629)	r_rb = −0.359	MWU	0.086	0.215
MLR	Before	0.242 ± 0.051	0.188 ± 0.055	—	—	WT	0.007	—
	After	0.180 [0.158–0.205]	0.189 [0.150–0.247]	—	—	MWU	0.720	—
	Within-group *p*	0.002	0.680	—	WBC + K: g_av = −0.936; KT: g_av = 0.113	WBC + K: PT; KT: PT	—	—
	Δ	−0.051 ± 0.053	0.007 ± 0.069	−0.058 (−0.102 to −0.014)	g = −0.927	WT	0.012	0.060
SII (×10^9^/L)	Before	522.467 [406.200–745.633]	603.104 [441.550–865.473]	—	—	MWU	0.559	—
	After	575.495 ± 303.965	671.263 ± 292.820	—	—	WT	0.371	—
	Within-group *p*	0.159	0.900	—	WBC + K: r_rb = −0.412; KT: r_rb = 0.044	WBC + K: WSR; KT: WSR	—	—
	Δ	−60.714 [−161.391–3.725]	25.692 [−123.226–143.933]	−81.031 (−230.236 to 118.219)	r_rb = −0.195	MWU	0.356	0.356
SIRI (×10^9^/L)	Before	0.962 [0.753–1.379]	0.667 [0.489–1.061]	—	—	MWU	0.030	—
	After	0.891 ± 0.417	0.786 ± 0.245	—	—	WT	0.393	—
	Within-group *p*	0.034	0.757	—	WBC + K: g_av = −0.512; KT: g_av = −0.090	WBC + K: PT; KT: PT	—	—
	Δ	−0.235 ± 0.402	−0.032 ± 0.409	−0.203 (−0.495 to 0.090)	g = −0.487	WT	0.168	0.280

Note: Values are presented as mean ± SD for comparisons performed using parametric tests and as median [IQR] for comparisons performed using non-parametric tests. Δ = post-treatment value − baseline value. Between-group differences in Δ were calculated as WBC + K minus KT. For change scores analyzed using Welch’s independent-samples *t*-test, effects are presented as mean differences with 95% CIs; for change scores analyzed using the Mann–Whitney U test, effects are presented as Hodges–Lehmann location-shift estimates with distribution-free 95% CIs. Negative values indicate a greater reduction or a smaller increase in the WBC + K group. Between-group *p*-values are unadjusted and two-sided. Effect sizes are reported for the main within-group and between-group change comparisons of NLR, PLR, MLR, SII, and SIRI. Hedges’ *g* was used for parametric independent-group comparisons, Hedges’ g_av for paired-samples *t*-tests, rank-biserial correlation (r_rb) for Mann–Whitney U tests, and matched-pairs rank-biserial correlations (r_rb) for Wilcoxon signed-rank tests. For between-group effects, positive values indicate a greater increase in WBC + K than in KT, whereas negative values indicate a greater reduction. For within-group effects, positive values indicate an increase, and negative values indicate a decrease from baseline. The q-values are reported only for the family of five between-group change-score comparisons for NLR, PLR, MLR, SII, and SIRI and were calculated using the Benjamini–Hochberg procedure. Within-group comparisons and analyses of component blood counts were supportive and descriptive and were not used to infer a differential treatment effect. Previously published hsCRP, BASDAI, and VAS pain outcomes are presented separately in Supplementary Table S1 as contextual outcomes. CI, confidence interval; IQR, interquartile range; KT, kinesiotherapy alone; MLR, monocyte-to-lymphocyte ratio; MWU, Mann–Whitney U test; NLR, neutrophil-to-lymphocyte ratio; PLR, platelet-to-lymphocyte ratio; PT, paired-samples *t*-test; SD, standard deviation; SII, systemic immune-inflammation index; SIRI, systemic inflammation response index; WBC + K, whole-body cryotherapy combined with kinesiotherapy; WSR, Wilcoxon signed-rank test; WT, Welch’s independent-samples *t*-test.

**Table 4:** Associations between changes in CBC-derived inflammatory indices and changes in hsCRP, BASDAI, and VAS pain.

Contextual Outcome	CBC-Derived Index	Spearman *r*_s_ (95% Bootstrap CI)	*p*-Value	*q*-Value
WBC + K group (*n* = 16)				
hsCRP	NLR	0.252 (−0.315 to 0.710)	0.347	0.950
hsCRP	PLR	−0.113 (−0.641 to 0.476)	0.676	0.950
hsCRP	MLR	−0.066 (−0.555 to 0.458)	0.807	0.950
hsCRP	SII	0.208 (−0.372 to 0.710)	0.441	0.950
hsCRP	SIRI	0.215 (−0.325 to 0.648)	0.424	0.950
BASDAI	NLR	0.416 (−0.173 to 0.857)	0.109	0.950
BASDAI	PLR	0.068 (−0.513 to 0.614)	0.803	0.950
BASDAI	MLR	0.175 (−0.330 to 0.639)	0.517	0.950
BASDAI	SII	0.283 (−0.319 to 0.777)	0.289	0.950
BASDAI	SIRI	0.364 (−0.181 to 0.771)	0.166	0.950
VAS pain	NLR	−0.017 (−0.520 to 0.557)	0.950	0.950
VAS pain	PLR	0.053 (−0.385 to 0.523)	0.846	0.950
VAS pain	MLR	−0.081 (−0.584 to 0.453)	0.767	0.950
VAS pain	SII	0.039 (−0.463 to 0.591)	0.887	0.950
VAS pain	SIRI	−0.113 (−0.594 to 0.475)	0.677	0.950
KT group (*n* = 16)				
hsCRP	NLR	−0.156 (−0.720 to 0.443)	0.564	0.853
hsCRP	PLR	−0.412 (−0.881 to 0.172)	0.113	0.848
hsCRP	MLR	−0.474 (−0.766 to −0.018)	0.064	0.848
hsCRP	SII	−0.285 (−0.712 to 0.265)	0.284	0.853
hsCRP	SIRI	−0.274 (−0.688 to 0.287)	0.305	0.853
BASDAI	NLR	−0.061 (−0.682 to 0.551)	0.824	0.853
BASDAI	PLR	0.195 (−0.423 to 0.697)	0.469	0.853
BASDAI	MLR	−0.081 (−0.601 to 0.496)	0.765	0.853
BASDAI	SII	0.050 (−0.559 to 0.653)	0.853	0.853
BASDAI	SIRI	−0.171 (−0.672 to 0.443)	0.525	0.853
VAS pain	NLR	−0.196 (−0.692 to 0.373)	0.467	0.853
VAS pain	PLR	−0.196 (−0.689 to 0.406)	0.467	0.853
VAS pain	MLR	−0.052 (−0.618 to 0.550)	0.848	0.853
VAS pain	SII	−0.091 (−0.606 to 0.464)	0.736	0.853
VAS pain	SIRI	−0.085 (−0.510 to 0.395)	0.755	0.853

Changes were calculated as post-treatment minus baseline values. CI, confidence interval; CBC, complete blood count; hsCRP, high-sensitivity C-reactive protein; BASDAI, Bath Ankylosing Spondylitis Disease Activity Index; VAS, visual analogue scale; FDR, false discovery rate; NLR, neutrophil-to-lymphocyte ratio; PLR, platelet-to-lymphocyte ratio; MLR, monocyte-to-lymphocyte ratio; SII, systemic immune-inflammation index; SIRI, systemic inflammation response index; WBC + K, whole-body cryotherapy combined with kinesiotherapy (*n* = 16); KT, kinesiotherapy alone (*n* = 16). The 95% CIs were obtained from 10,000 non-parametric within-group bootstrap resamples. The Benjamini–Hochberg procedure was applied separately to the family of 15 correlations within each treatment group. Because the procedure enforces monotonicity across the ordered adjusted *p*-values, different unadjusted *p*-values may yield identical *q*-values. Consequently, all 15 comparisons in the WBC + K group yielded *q* = 0.950, whereas repeated values of *q* = 0.848 and *q* = 0.853 occurred in the KT group. No correlation remained statistically significant after FDR correction.

## Data Availability

The de-identified individual-level data and analysis code necessary to reproduce the derived indices and statistical analyses are available from the corresponding author upon reasonable request, subject to applicable ethical and data-protection requirements.

## Supplementary Materials

The following supporting information can be downloaded at https://www.mdpi.com/article/10.3390/jcm15207894/s1, Table S1: Previously published hsCRP, BASDAI, and VAS pain outcomes presented as contextual variables; Table S2: Exploratory associations between post-treatment CBC-derived inflammatory indices and post-treatment hsCRP, BASDAI, and VAS pain.

## Abbreviations

The following abbreviations are used in this manuscript:

AS	Ankylosing spondylitis
ASAS	Assessment of SpondyloArthritis International Society
ASDAS	Ankylosing Spondylitis Disease Activity Score
axSpA	Axial spondyloarthritis
BASDAI	Bath Ankylosing Spondylitis Disease Activity Index
bDMARDs	Biological disease-modifying antirheumatic drugs
BMI	Body mass index
CRP	C-reactive protein
csDMARDs	Conventional synthetic disease-modifying antirheumatic drugs
EDTA	Ethylenediaminetetraacetic acid
EULAR	European Alliance of Associations for Rheumatology
HLA-B27	Human leukocyte antigen B27
hsCRP	High-sensitivity C-reactive protein
KT	Kinesiotherapy alone
MLR	Monocyte-to-lymphocyte ratio
NLR	Neutrophil-to-lymphocyte ratio
NSAIDs	Non-steroidal anti-inflammatory drugs
PLR	Platelet-to-lymphocyte ratio
SD	Standard deviation
SII	Systemic immune-inflammation index
SIRI	Systemic inflammation response index
VAS	Visual analogue scale
WBC	Whole-body cryotherapy
WBC + K	Whole-body cryotherapy combined with kinesiotherapy
