# Supplementary material for: Complete Blood Count-Derived Inflammatory Indices Following Whole-Body Cryotherapy Combined with Kinesiotherapy Versus Kinesiotherapy Alone in Men with Ankylosing Spondylitis
Source: J Clin Med. 2026 Oct 13;15(20):7894. doi: 10.3390/jcm15207894 (PMC13705415; doi:10.3390/jcm15207894)
Supplement: Supplementary file 1 [file jcm-15-07894-s001.zip › Supplemetary Table S1.pdf]

Supplementary Table S1. Previously published hsCRP, BASDAI, and VAS pain outcomes presented as contextual variables.

Outcome	Assessment	WBC + K group	KT group	Between-group difference in Δ (95% CI)	Effect size	Exact test	*p*-value
**BASDAI**	Before	5.430 ± 1.610	5.280 ± 1.710	—	—	WT	0.792
	After	3.290 ± 0.910	4.530 ± 1.620	—	—	WT	0.014
	Within-group p	<0.001	<0.001	—	WBC + K: g_av = −1.593; KT: r_rb = −1.000	WBC + K: PT; KT: WSR	—
	Δ	−1.900 [−2.950 to −1.470]	−0.60 [−0.820 to −0.570]	−1.100 (−2.100 to −0.800)	r_rb = −0.707	MWU	<0.001
**hsCRP (mg/L)**	Before	9.350 [2.270 to 15.930]	9.000 [3.300 to 15.350]	—	—	MWU	0.806
	After	2.750 [0.750 to 11.380]	7.000 [3.020 to 17.680]	—	—	MWU	0.175
	Within-group p	0.002	0.632	—	WBC + K: r_rb = −0.882; KT: r_rb = 0.147	WBC + K: WSR; KT: WSR	—
	Δ	−3.650 [−5.850 to −0.800]	0.400 [−0.870 to 2.120]	−3.500 (−6.100 to −1.100)	r_rb = −0.574	MWU	0.006
**VAS pain (points)**	Before	5.250 ± 1.730	5.000 ± 1.630	—	—	WT	0.676
	After	3.000 [1.380 to 4.000]	4.000 [2.750 to 5.000]	—	—	MWU	0.072
	Within-group p	<0.001	0.001	—	WBC + K: r_rb = −1.000; KT: r_rb = −0.978	WBC + K: WSR; KT: WSR	—
	Δ	−1.500 [−2.250 to −1.000]	−1.000 [−1.000 to −0.750]	−1.000 (−2.000 to 0.000)	r_rb = −0.449	MWU	0.021

Note: Values are presented as mean ± SD for comparisons performed using parametric tests and as median [IQR] for comparisons performed using non-parametric tests. Δ = post-treatment value − baseline value. Between-group differences in Δ were calculated as WBC + K minus KT and are presented as Hodges–Lehmann

location-shift estimates with distribution-free 95% CIs. All p-values are unadjusted and two-sided and are presented for descriptive contextual purposes; these outcomes were not included in the multiplicity family defined for the five CBC-derived indices. Effect sizes are reported for the main within-group and between-group change comparisons. Hedges' g_av was used for paired-samples t-tests, rank-biserial correlation (r_rb) for Mann–Whitney U tests, and matched-pairs rank-biserial correlation (r_rb) for Wilcoxon signed-rank tests. Because of the discrete distribution and tied observations, the distribution-free confidence interval for the Hodges–Lehmann estimate may not correspond exactly to the Mann–Whitney U test p-value. For between-group effects, negative values indicate a greater reduction in WBC + K than in KT; for within-group effects, negative values indicate a decrease from baseline. The outcomes in this table were reported previously and were reanalysed from the original individual-level data using the statistical decision rules specified for the present secondary analysis. In the earlier analysis, the post-treatment VAS comparison was performed using Welch's independent-samples t-test (p = 0.047); because the revised analysis identified a non-normal distribution, the Mann–Whitney U test was used here (p = 0.072). BASDAI, Bath Ankylosing Spondylitis Disease Activity Index; CI, confidence interval; hsCRP, high-sensitivity C-reactive protein; IQR, interquartile range; KT, kinesiotherapy alone; MWU, Mann–Whitney U test; PT, paired-samples t-test; SD, standard deviation; VAS, visual analogue scale; WBC + K, whole-body cryotherapy combined with kinesiotherapy; WSR, Wilcoxon signed-rank test; WT, Welch's independent-samples t-test.
