# Supplementary material for: Complete Blood Count-Derived Inflammatory Indices Following Whole-Body Cryotherapy Combined with Kinesiotherapy Versus Kinesiotherapy Alone in Men with Ankylosing Spondylitis
Source: J Clin Med. 2026 Oct 13;15(20):7894. doi: 10.3390/jcm15207894 (PMC13705415; doi:10.3390/jcm15207894)
Supplement: Supplementary file 1 [file jcm-15-07894-s001.zip › Suplementary Table S2.pdf]

Supplementary Table S2. Exploratory associations between post-treatment CBC-derived inflammatory indices and post-treatment hsCRP, BASDAI, and VAS pain.

Contextual outcome	CBC-derived index	Spearman r_s (95% bootstrap CI)	p	q
**WBC + K group (n = 16)**				
hsCRP	NLR	0.121 (-0.435 to 0.871)	0.656	0.703
hsCRP	PLR	0.274 (-0.298 to 0.808)	0.305	0.508
hsCRP	MLR	0.143 (-0.426 to 0.734)	0.598	0.690
hsCRP	SII	0.236 (-0.332 to 0.890)	0.380	0.530
hsCRP	SIRI	0.231 (-0.334 to 0.872)	0.389	0.530
BASDAI	NLR	0.393 (-0.213 to 0.816)	0.132	0.248
BASDAI	PLR	0.495 (0.005 to 0.809)	0.051	0.154
BASDAI	MLR	0.566 (0.165 to 0.790)	0.022	0.154
BASDAI	SII	0.393 (-0.131 to 0.753)	0.132	0.248
BASDAI	SIRI	0.499 (-0.021 to 0.853)	0.049	0.154
VAS pain	NLR	0.620 (0.156 to 0.926)	0.010	0.154
VAS pain	PLR	0.181 (-0.317 to 0.650)	0.503	0.628
VAS pain	MLR	0.024 (-0.543 to 0.574)	0.929	0.929
VAS pain	SII	0.498 (0.065 to 0.785)	0.050	0.154
VAS pain	SIRI	0.401 (-0.108 to 0.776)	0.124	0.248
**KT group (n = 16)**				
hsCRP	NLR	0.150 (-0.402 to 0.622)	0.579	0.978
hsCRP	PLR	0.007 (-0.517 to 0.543)	0.978	0.978
hsCRP	MLR	-0.549 (-0.848 to -0.063)	0.028	0.413
hsCRP	SII	0.467 (-0.033 to 0.846)	0.068	0.512
hsCRP	SIRI	-0.046 (-0.533 to 0.441)	0.867	0.978
BASDAI	NLR	-0.150 (-0.654 to 0.380)	0.579	0.978
BASDAI	PLR	-0.013 (-0.518 to 0.544)	0.961	0.978
BASDAI	MLR	0.140 (-0.362 to 0.568)	0.606	0.978
BASDAI	SII	-0.222 (-0.699 to 0.343)	0.408	0.978
BASDAI	SIRI	0.112 (-0.461 to 0.639)	0.680	0.978
VAS pain	NLR	0.121 (-0.412 to 0.602)	0.655	0.978
VAS pain	PLR	0.072 (-0.502 to 0.626)	0.792	0.978
VAS pain	MLR	-0.233 (-0.655 to 0.281)	0.385	0.978
VAS pain	SII	0.151 (-0.452 to 0.692)	0.577	0.978
VAS pain	SIRI	-0.018 (-0.573 to 0.519)	0.947	0.978

Correlations were calculated using post-treatment values. CI, confidence interval; CBC, complete blood count; hsCRP, high-sensitivity C-reactive protein; BASDAI, Bath Ankylosing Spondylitis Disease Activity Index; VAS, visual analogue scale; FDR, false discovery rate; NLR, neutrophil-to-lymphocyte ratio; PLR, platelet-to-lymphocyte ratio; MLR, monocyte-to-lymphocyte ratio; SII, systemic immune-inflammation index; SIRI, systemic inflammation response index; WBC + K, whole-body cryotherapy combined with kinesiotherapy (n = 16); KT, kinesiotherapy alone (n = 16). The 95% CIs were obtained from 10,000 nonparametric within-group

bootstrap resamples. Unadjusted *p*-values are two-sided. Benjamini–Hochberg-adjusted *q*-values were calculated from the unrounded *p*-values separately for the family of 15 correlations within each treatment group. Because the Benjamini–Hochberg step-up procedure enforces monotonicity across the ranked tests, different unadjusted *p*-values may yield identical adjusted *q*-values. All *p*- and *q*-values are reported to three decimal places. Because the bootstrap confidence intervals and unadjusted p-values were obtained using different statistical procedures, their boundaries may not correspond exactly to the nominal significance threshold.
